# Towards practical privacy-preserving genome-wide association study

**DOI:** 10.1186/s12859-018-2541-3

**Published:** 2018-12-20

**Authors:** Charlotte Bonte, Eleftheria Makri, Amin Ardeshirdavani, Jaak Simm, Yves Moreau, Frederik Vercauteren

**Affiliations:** 1 imec-COSIC, Department of Electrical Engineering, KU Leuven, Leuven, Belgium; 2 ABRR, Saxion University of Applied Sciences, Enschede, The Netherlands; 3 STADIUS KU Leuven, Leuven, Belgium

**Keywords:** Genome-wide association study (GWAS), Homomorphic encryption (HE), Secure multiparty computation (MPC)

## Abstract

**Background:**

The deployment of Genome-wide association studies (GWASs) requires genomic information of a large population to produce reliable results. This raises significant privacy concerns, making people hesitate to contribute their genetic information to such studies.

**Results:**

We propose two provably secure solutions to address this challenge: (1) a somewhat homomorphic encryption (HE) approach, and (2) a secure multiparty computation (MPC) approach. Unlike previous work, our approach does not rely on adding noise to the input data, nor does it reveal any information about the patients. Our protocols aim to prevent data breaches by calculating the *χ*^2^ statistic in a privacy-preserving manner, without revealing any information other than whether the statistic is significant or not. Specifically, our protocols compute the *χ*^2^ statistic, but only return a yes/no answer, indicating significance. By not revealing the statistic value itself but only the significance, our approach thwarts attacks exploiting statistic values. We significantly increased the efficiency of our HE protocols by introducing a new masking technique to perform the secure comparison that is necessary for determining significance.

**Conclusions:**

We show that full-scale privacy-preserving GWAS is practical, as long as the statistics can be computed by low degree polynomials. Our implementations demonstrated that both approaches are efficient. The secure multiparty computation technique completes its execution in approximately 2 ms for data contributed by one million subjects.

## Background

The goal of a genome-wide association study (GWAS) is to identify genetic variants that are associated with traits. Large-scale sequencing provides reliable information on single nucleotide variants (SNVs). To date, researchers worked mostly on identifying genetic alterations which lead to classification of SNVs and SNPs (single nucleotide polymorphims). Therefore, when we mention SNVs we refer to both frequent SNPs, and less frequent SNVs. A common approach is to divide the population into a disease, and a healthy group based on whether the individual has the particular disease. Each individual gives a sample DNA from which millions of genetic variants (i.e., SNVs) are identified. If a variant is more frequent in individuals with the disease, it will likely be associated with the specific genetic disorder and be classified as a potential marker of the disease.

### Motivation for the distributed setup with secure computations

Having a large population size is crucial for GWAS, because it allows to improve the accuracy of identified associations, especially for *rare* genetic disorders. Two recent developments result in a significant increase of the available data for GWAS: First, the development of cheap next generation sequencing (NGS). Second, the creation of *distributed genomic databases*, which enable pooling of data from many hospitals, and research centers, further increasing the population sizes of the studies by 10-50 times. Several such distributed databases have recently been proposed, including NGS-Logistics [1], Elixir, and GA4GH Beacon [2].

In studies like GWAS, which use personally identifiable genetic markers of the participants as input, the privacy of the patients and protection of their sensitive data becomes of great importance. It has been shown by Malin et al. [3], that releasing the raw data even after removal of explicit identifiers, does not protect an individual from getting identified. The classical approach to solve this privacy problem involves a trusted third party who first collects both the SNV, and the trait data, then carries out the statistical test, and finally either a) only reveals the very few SNVs that have statistically significant association or b) reveals all aggregate data on SNVs but masks them with sufficient noise to guarantee *differential privacy*. For example, previous works by Uhlerop et al. [4], and by Simmons and Berger [5] have focused on computing a differentially private *χ*^2^ test. However, setting up such a trusted third party has significant legal, and technical difficulties given the sensitive nature of the underlying data.

The aforementioned privacy concerns make both individuals and medical centers hesitant to share this private data. Hence, centralized (third party) datasets collected for research purposes remain small. Our goal is to address this challenge, in a way that the data can be shared without trusting an external third party. In our setup, the medical centers aggregate and encrypt or secret share the patient data before sending it to a third party for research purposes. This ensures the privacy of the input data, because the only party with access to the raw input data is the medical center which gathers it. Hence, our distributed solution allows to combine input data from different medical centers to construct a large dataset for research, while eliminating the privacy implications. Our solution can even scale up to *millions of patients*, and perform *millions or tens of millions of hypothesis tests* per day. This enables the first step towards large-scale distributed GWAS, where multiple medical centers contribute data, without relying on a trusted third party. Such large data collections would also allow association studies on rare diseases.

Another reason to opt for our secure computation solution instead of the trusted third party one, is that the latter does not provide defense against malicious agents or operating system bugs, which might result in leakage of information. In our case, such a mishap would reveal encrypted values (or shares of a value, resp.), which essentially provides no information to the adversary, as long as the secret key is not comprimised (or the adversary has fewer than *n* shares, resp.).

### Motivation for the yes/no response

Studies related to GWAS raised even more privacey concerns. Research has brought to light that releasing aggregated statistics related to GWASs leaks information in an implicit way. Therefore, it is not enough to protect only the input data; care has to be taken when releasing aggregated results to the public, as well. The work of Homer et al. [6] showed that the presence of an individual in the case group can be determined from the aggregated allele frequencies. One can argue that this attack requires an adversary to have at least 10,000 SNVs from the victim. However, we assume that with the current sequencing techniques, this is no longer a challenge and hence Homer’s attack is posing a real threat nowadays. By computing with encrypted or secret shared data, and only revealing a boolean value indicating significance, we prevent adversaries from obtaining the aggregated allele frequencies, thus protecting against Homer’s attack.

Shortly following Homer’s attack, Wang et al. [7] reported an attack based on statistical values reported in GWAS papers. Even though, the attack of Wang et al. [7] requires more statistical data than what our solution would reveal, such developments show that we need to be careful with the amount of information we publish. Our solution anticipates future statistical attacks, by not publishing any statistic values at all.

### Additional properties of the our setup

Our proposal consists of a cryptographic approach, where the trusted third party performing research is replaced by a *privacy-preserving* system, which receives the input in encrypted (protected) form from a set of distributed parties (e.g., hospitals), performs the *χ*^2^ test, and only publicly discloses whether the current test is significant or not. Since nothing except the final answer is revealed during the execution of our protocols, the proposed system enjoys various security guarantees, even against malicious agents who gain access to the servers executing the system.

Even though the aforementioned attacks show it is not a good idea to reveal the *χ*^2^ value, the value itself would be highly interesting for research purposes. Therefore, it is worth mentioning that our current system can be easily adapted to return the significance value itself. However, since revealing the values can cause privacy issues, we suggest to incorperate an authentication process to the system if the *χ*^2^ value should be revealed. This way the access to the actual *χ*^2^ values can be restricted to authenticated users. As such, the researchers can have access to the actual result, while it stays hidden from the public and therefore cannot be abused in an attack like the aforementioned ones.

By only revealing the yes/no answer, our system indicates whether the SNV is a possible marker. To determine whether or not this SNV is actually causally linked to the disease more statistics need to be computed. Therefore, we assume that for the selected SNVs –indicated by our system– the researcher would request specific patient data from the different centers for further analysis. We assume this will happen with the current techniques for requesting data for GWASs. However, we expect patients to be more inclined to share their data for research, even despite the potential privacy concerns, when the researchers explain to them, with the aid of the public tables, that their data is highly relevant for the study of a specific disease.

Additionally, it is common practice in GWASs, and more general bioinformatics studies to publish only when significant results are found. This means that all the insignificant (yet identified) results are not published, despite the fact that they could also contribute in finding, or eliminating interesting correlations. In fact, a non-significant correlation between a genotype and a phenotype can serve as a proof that a certain mutation is not related to a disease. Our solution comes to bridge this gap, as we aim to construct a public table, listing all possible mutations, versus all possible phenotypes, and indicating whether the initial relationship between them (indicated by the *χ*^2^ test) is significant or not. By publishing also the insignificant results in our public table, mutations not related to phenotypes can be immediately shown, allowing the researchers to discard them, and focus only on the significant ones.

To allow for a privacy-preserving system addressing our challenges, we propose two secure approaches: one based on homomorphic encryption (HE), and one based on multiparty computation (MPC). We also compare their security guarantees, and their efficiency in terms of execution time of practical implementations. Homomorphic encryption refers to a set of cryptographic tools that allow certain computations to take place in the encrypted domain, while the resulting ciphertext, when decrypted, is the expected (correct) result of operations on the plaintext data. Secure multiparty computation aims at allowing a similar functionality, amongst several mutually distrusting parties, who wish to compute a function without revealing their private inputs. With the latter approach, communication between the computing parties is required for the execution of the cryptographic protocols.

In the MPC setting, there are two main security models used, offering passive, or active security, respectively. *Passive security*, also known as security in the semi-honest model, assumes that the protocol participants are honest-but-curious. This means that they are trying to collect as much information as possible from the protocol execution, but they do follow the protocol instructions honestly. *Active security*, also known as malicious security, offers stronger security guarantees, assuming that adversaries or corrupted protocol participants may arbitrarily deviate from the protocol instructions. In both security models, we can build protocols assuming an honest majority of the protocol participants, or a dishonest majority. Our solution with MPC offers the highest security guarantees being built in the malicious model, with dishonest majority.

Specifically, we make the following contributions:

- We propose the first *somewhat homomorphic encryption* approach to withstand GWAS attacks such as the ones described by Homer et al. [6].
- We develop a *multiparty computation* solution for GWAS that is efficient for realistic sample sizes.
- We propose a new masking technique to allow efficient secure comparisons.
- We compare the security, and efficiency of HE and MPC on a real-life application.
- We demonstrate the practicality of our solutions, based on their short running times, which are in the range of 1.9-2.4 ms for the MPC approach.
- We show that our solution scales logarithmically in the number of subjects contributing their genetic information, allowing us to treat current population sizes, and being able to scale to larger (future) GWASs for millions of people.

### Related work

#### Homomorphic encryption approach

There has already been some work on using homomorphic encryption to preserve the privacy of the patients while performing statistics on genome data. Kim et al. [8] present the computation of minor allele frequencies, and the *χ*^2^ statistic with the use of the homomorphic BGV and YASHE encryption schemes. They use a specific encoding technique to improve on the work of Lauter et al. [9]. However, they only compute the allele counts homomorphically, and execute the other operations on the decrypted data. Another work on GWASs using fully homomorphic encryption was published by Lu et al. [10]. They also start from encrypted genotype/phenotype information that is uploaded to a cloud for each person separately. Then they perform the minimal operations necessary to provide someone with access to the decryption key with the necessary values to construct the contingency table for the requested case based on the data present on the cloud. Hence, when performing a request, the scientist gets three encrypted values, and based on those he can, after decryption, reconstruct the contingency table, and compute the *χ*^2^ statistic in the clear. These solutions are not resistant to attacks like the one described by Homer et al. [6]. Our solution improves on these previous works by performing the *χ*^2^ computation in the encrypted domain, and revealing only whether or not the *χ*^2^ value is significant for this case, which makes the previously mentioned attacks impossible.

Sadat et al. [11] propose a hybrid system called SAFETY, to compute various statistical values over genomic data. This hybrid system consists of a combination of the partially homomorphic Paillier scheme with the secure hardware component of Intel Software Guard Extensions (Intel SGX) to ensure both high efficiency, and privacy. With this hybrid system they propose a more efficient way to get the total counts of all patients for a specific case. By using the additive property of the homomorphic Paillier scheme, they reduce the computational overhead of decrypting all individual encrypted outputs received from the different servers. Afterwards it uses the Intel SGX component to perform the *χ*^2^ computations. Even though, the results of this system scale well for increasing number of servers that provide data for the computation, the system does not provide the same functionality as our solution. Sadat et al. [11] mention that the only privacy guarantee for the final computation result against the attack described by Homer et al. [6] is the assumption that the researcher decrypting the result is semi-honest. This is the main difference with our work: with our solution only the significance of the test will be made public. As mentioned before, the current system can be easily adapted to return the *χ*^2^ value itself but due to known attacks we want to avoid making these values public. Hence, we believe that if our system is adapted to reveal the *χ*^2^ values, it should only reveal these values after authentication of the requesting party.

Zhang et al. [12], construct an algorithm, which performs the whole *χ*^2^ statistic in the homomorphic domain. To compute the division, they construct a lookup table in which they link the result of their computation with the nominator and denominator of the corresponding, simplified fraction. Therefore, an authenticated user can look up the correct fraction in the lookup table after decrypting the result, and hence recover the result of the *χ*^2^ statistic. Even though their strategy performs well, it does not scale enough to treat the large datasets we envision in our application. Increasing the number of patients in the study would increase the circuit depth significantly, which comes with several disadvantages including increasing the parameter sizes, and hence the key size, and ciphertexts size, as well as the computation time.

#### Secure multiparty computation approach

Kamm et al. [13] propose a solution to address the privacy challenges in genome-wide association studies. Their application scenarios, much like ours, focus on large data collections from several biobanks, and their solutions are based on the same fundamental techniques as ours. However, the setting of Kamm et al. [13] requires all raw genotype, phenotype, and clinical data to be entered to the secure shared database. To the contrary, our setting assumes that only the aggregate values, necessary to identify the significance of a gene-disease relationship (i.e., the contingency tables recording the counts of genotypes vs. phenotypes), are contributed by each biobank. This is a simpler, and more realistic setting, which not only is likely to be implemented in the near future, but also alleviates the computational cost of the proposed solutions. Unlike the approach of Kamm et al. [13], and the alternatives that they suggest, our solution achieves active security with dishonest majority (contrary to the semi-honest security suggested). This means that our protocols tolerate dishonest behavior by the majority of the computing parties, while preserving privacy, and still guarantee the correctness of accepted results. Kamm et al.’s protocols assume that the computing parties –the biobanks– cannot be corrupted, which we consider to be a strong assumption.

Independent and concurrent work by Cho et al. [14] tries to address the same problem as we do in our work, using multiparty computation techniques. They focus on a method that enables the identification and correction for population biases before computing the statistics. However, just like the work of Kamm et al. [13], they make the strong assumption of semi-honest security. In practice, the semi-honest security is not a sufficient security guarantee for GWAS, as attackers who have obtained access to the systems are likely to employ active measures to obtain the data.

Constable et al. [15] present a garbled-circuit based MPC approach to perform GWAS. Their solution can compute in a privacy-preserving manner the minor allele frequency (MAF), and the *χ*^2^ statistic. Similarly to the work of Kamm et al. [13], the framework of Constable et al. [15] requires the raw genotype, and phenotype data, increasing the workload of the proposed privacy-preserving system. In contrast to our solution, which can scale to hundreds of medical centers contributing data to the GWAS, the solution of Constable et al. [15] only works for two medical centers. Despite the strong security guarantees that our approach offers, which generally presents itself as a tradeoff to efficiency, our proposal is faster than that of Constable et al. [15]. This is also due to the fact that we have optimized the computations of the *χ*^2^ statistic, in such a way that the expensive computations in the privacy-preserving domain, are avoided to the maximum extent possible.

Zhang et al. [16] propose a secret-sharing based MPC approach to solve the same GWAS problem as Constable et al. [15]. Although Zhang et al.’s solution can scale to more than two medical centers contributing data to the GWAS, the approach has the same inherent limitations (e.g., requiring raw genomic data as input) that their application scenario incurs. The works of Zhang et al. [16], Constable et al. [15], and Cho et al. [14] have not considered protecting the aggregate statistic result of the private computation, which –as Homer et al. [6] showed– can be used to breach an individual’s privacy. We additionally protect the aggregate statistic result, while at the same time allowing for a public list to be created, showing which SNVs are significant for a certain disease.

## Methods

### Distributed GWAS scenario

In this paper we aim at identifying which mutations are linked to which phenotypes, without compromising the privacy of the patients. Specifically, there are *K* centers (hospitals) who each have genotype (SNV), and phenotype (trait) data. For a single genotype-phenotype pair a center *k* has a 2×2 contingency table^1^ of the counts of patients for all 4 possible combinations of genotype, and phenotype (see Table 1). The goal is to perform a privacy-preserving computation that adds together all contingency tables from individual centers, then computes the Pearson’s *χ*^2^ test statistic [17], and finally reveals a boolean value indicating whether the computed statistic is larger than a predetermined significance threshold *t*. This threshold is chosen based on the *p*-value, and the correction for multiple hypothesis testing. For example, using significance level 0.01 with Bonferroni correction for 10 million tests results in *t*=37.3, and for 100 million tests *t*=41.8.

**Table 1:** Representation of a contingency table containing the number of observed genotypes *i* per phenotype *j* noted by *O*_*i*,*j*_

	*phenotype*	¬*phenotype*	
*genotype*	*O*_1,1_	*O*_1,2_	*RT*_1_ = *O*_1,1_+*O*_1,2_
¬*genotype*	*O*_2,1_	*O*_2,2_	*RT*_2_ = *O*_2,1_+*O*_2,2_
	*CT*_1_ = *O*_1,1_ + *O*_2,1_	*CT*_2_ = *O*_1,2_ + *O*_2,2_	*N* = *CT*_1_ + *CT*_2_ = *RT*_1_ + *RT*_2_

In the table we also calculate the Row Totals (*RT*_*i*_), Column Totals (*CT*_*j*_), as well as the grand total (*N*)

We propose two different methods for carrying out the *χ*^2^ test without disclosing the input, and intermediate values. The first method performs all computations on *homomorphically* encrypted data, while the second applies techniques of *secure multiparty computation* to achieve the same goal. Both methods follow the same general outline, presented below. The first step is to encrypt (or secret share) all the input tables from the centers, and securely compute the aggregate contingency table

1 $$ O_{ij} = \sum\limits_{k=1}^{K} O_{ij}^{(k)},  $$

where $O_{ij}^{(k)}$ is the data from *k*-th center. This step is straightforward in both methods.

Next to determine the significance of the relation between a mutation, and a phenotype, we calculate the Pearson’s *χ*^2^ test statistic [17] on the aggregated contingency table *O*, and check whether this value is above the threshold *t*. The Pearson’s *χ*^2^ statistic is given by the following formula:

2 $$ \chi^{2} = \sum\limits_{i,j \in \{1,2\}}\frac{\left(O_{ij} - {model}_{ij}\right)^{2}}{{model}_{ij}},  $$

where *model*_*ij*_=(*RT*_*i*_·*CT*_*j*_)/*N* with *RT*_*i*_=*O*_*i*,1_+*O*_*i*,2_ being the row total, *CT*_*j*_=*O*_1,*j*_+*O*_2,*j*_ being the column total, and *N* the total number of patients.

Since division is a costly operation in both the homomorphic domain, and secret shared domain, we will rewrite the formula of the *χ*^2^ statistic as follows:

3 $$  {\begin{aligned} \chi^{2} \,=\, \frac{{RT}_{1} \cdot {CT}_{1} \cdot \left(N \cdot O_{2,2} - {RT}_{2} \cdot {CT}_{2}\right)^{2}} {N \cdot {RT}_{1} \cdot {RT}_{2} \cdot {CT}_{1} \cdot {CT}_{2}} \,+\, \frac{{RT}_{1} \cdot {CT}_{2} \cdot \left(N \cdot O_{2,1} - {RT}_{2} \cdot {CT}_{1}\right)^{2}} {N \cdot {RT}_{1} \cdot {RT}_{2} \cdot {CT}_{1} \cdot {CT}_{2}} \\ \,+\, \frac{{RT}_{2} \cdot {CT}_{1} \cdot \left(N \cdot O_{1,2} - {RT}_{1} \cdot {CT}_{2}\right)^{2}} {N \cdot {RT}_{1} \cdot {RT}_{2} \cdot {CT}_{1} \cdot {CT}_{2}} \,+\, \frac{{RT}_{2} \cdot {CT}_{2} \cdot \left(N \cdot O_{1,1} - {RT}_{1} \cdot {CT}_{1}\right)^{2}} {N \cdot {RT}_{1} \cdot {RT}_{2} \cdot {CT}_{1} \cdot {CT}_{2}}. \end{aligned}}  $$

As a final step, we need to compare whether *χ*^2^≥*t*. To do that, we calculate the numerator, and denominator of the fraction in Eq. (3), separately. Subsequently, we multiply the denominator of the fraction with the threshold value *t*, and finally check inequality (4), without revealing any of the private inputs in the contingency tables.

4 $$ {\begin{aligned} {}{RT}_{1} \cdot {CT}_{1} \cdot \left(N \cdot O_{2,2} - {RT}_{2} \cdot {CT}_{2}\right)^{2} + {RT}_{1} \cdot {CT}_{2} \cdot \left(N \cdot O_{2,1} - {RT}_{2} \cdot {CT}_{1}\right)^{2} \\ {}+ {RT}_{2} \cdot {CT}_{1} \cdot \left(N \cdot O_{1,2} - {RT}_{1} \cdot {CT}_{2}\right)^{2} + {RT}_{2} \cdot {CT}_{2} \cdot \left(N \cdot O_{1,1} - {RT}_{1} \cdot {CT}_{1}\right)^{2} \\ \overset{?}\geq t \cdot \left(N \cdot {RT}_{1} \cdot {RT}_{2} \cdot {CT}_{1} \cdot {CT}_{2}\right). \end{aligned}}  $$

This computation is repeated for every phenotype-genotype pair, and the results are aggregated in a public table indicating whether a mutation is significant for a particular phenotype, or not. Since the price of DNA sequencing has decreased a lot, we assume new data will keep becoming available. Taking this new data into account for the computation of our public table, requires running our protocols anew, and it will change the table results. Therefore, we propose to make the table dynamic. There will be a fixed time interval, which allows the centers to gather more data and include this data in their contingency tables. The new table values will then be encrypted/secret shared and the computation of the fresh public table will be executed, after which the new results will be published.

#### Efficient masking-based comparison

To the best of our knowledge the state-of-the-art techniques to perform secure comparisons, both in the homomorphic, and in the secret shared domain, require bitwise operations on the secret inputs, which have a high total cost. To allow for a practically efficient implementation of our solution, we consider a masking technique to perform the comparison instead of the bit-decomposition of our inputs. By masking the values we need to compare, we can later securely reveal the masked result upon decryption, since the mask will hide the original secret value. Hence, masking allows us to perform the comparison without revealing the values we want to keep secret. Comparing two values *x* and *y* can be done by comparing their difference with zero. Our mask for the value *x*−*y* consists of multiplying this value with a positive random number. We require the multiplier to be positive to preserve the original relation of our difference *x*−*y* with zero. The second step is adding another random number (different than the previous one) to the already multiplied result. We require this random number to be smaller than the first one, again to preserve the original relation with zero. Let us denote the masked difference with $\widehat {x-y}$, then for two positive random numbers *r* and *r*^′^, with *r*^′^ in the range [1,*r*), our proposed masking is given by $\widehat {x-y}=r\cdot (x-y)+r'$. In our setup we are working with homomorphic (or secret shared) values, so this masking has to be performed on encrypted (or secret shared) values. For an integer *x*, we denote [ [*x*] ] either the homomorphic encryption of *x* or its secret shared value. Masking in the homomorphic or secret shared domain will then be computed as $[\!\![\widehat {x-y}]\!\!] = [\!\![r]\!\!] \cdot [\!\![x-y]\!\!] + [\!\![r']\!\!]$, with *r* and *r*^′^ random numbers satisfying the following condition: *r* is selected to be a positive integer number (bounded properly so as to fit the largest possible input sizes our framework can handle), and then *r*^′^ is randomly selected in the range [ 1,*r*) (i.e., such that *r*^′^<*r*). Afterwards the masked value is revealed by respectively decrypting or opening the calculated value. Depending on the sign of $(\widehat {x-y})$ we can deduce the relationship between *x* and *y* (i.e., if $(\widehat {x-y}) > 0$ then *x*>*y*, otherwise *x*<*y*).

Given properly selected *r*, and *r*^′^, the correctness of this masking-based comparison is straightforward. To ensure preservation of the security and correctness of the masking, we require one of the medical centers to properly select *r*, and *r*^′^ within suitable bounds. Note that this requirement does not increase the level of trust we need to put in the medical centers (nor does it reduce the security of the system). We already trust the medical centers to provide our privacy-preserving system with their correct inputs. Upon selection of the values *r*, and *r*^′^, the medical center in question homomorphically encrypts these values, or secret shares them to the computation servers, along with its own contributed inputs.

The proposed type of masking, which allows us to perform the comparison, could leak information about the secret input to the inequality, which in our case is the difference *x*−*y*, when the system is queried multiple times with the same input. However, in our scenario, the proposed system cannot be queried at will. We suggest the calculation of a table listing all possible phenotypes, and all possible mutation positions, which will become public. This table will be computed on updated input data after a fixed time interval. While constructing the table we select *r* and *r*^′^ at random for each contingency table, thus the random values *r* and *r*^′^ will only be used once with certain input values. After the fixed time interval, we expect the input values to be changed, so we repeat the whole setup and select new random values for each contingency table. Hence, by recomputing the table at fixed times and not allowing users to query, we ensure that no information is leaked by our system.

Let us for completeness briefly discuss the leakage that occurs if a party observing the masked result of the inequality check can submit multiple queries on the same inputs and obtain the masked values for these queries. If this would be possible, an adversary would be able to approximate the value of *x*−*y* from the obtained list of masked values. The maximum of this observed list will be close to the bound set for the randomness *r* times the difference *x*−*y*. Hence, by deduction, if we divide the maximum observed masked value by the upper bound on *r*, we will get a good approximation of the value *x*−*y*.

In the event of a malicious party being able to observe the intermediate values revealed by our approach (i.e., the value of the masked difference), and given that this malicious party can trigger multiple computations of the same table entry, one can prevent the aforementioned leakage by selecting the random values *r* and *r*^′^ once per table entry, and keep them thereafter fixed, until the actual inputs to the protocol (contributed by each medical center) change.

Our general approach that applies both to the HE, and the MPC setting is detailed in Algorithm 1. For the technical details on the HE, and MPC methods used, as well as the implementation details of our proposal, we refer the reader to the full version of our paper [18]. For the MPC setting we performed experiments both with the proposed masking-based comparison, and with the standard, bit-decomposition based comparison, as implemented in SPDZ-2 [19]. Our experiments showed that for the online MPC protocol, the performance difference between the secure comparison, and the masking-based comparison is negligible (i.e., 0.6 ms CPU time difference, and ∼5kB communication cost difference). Thus, we opted for the slightly less efficient bit-decomposition based comparison, since it is available in SPDZ-2.


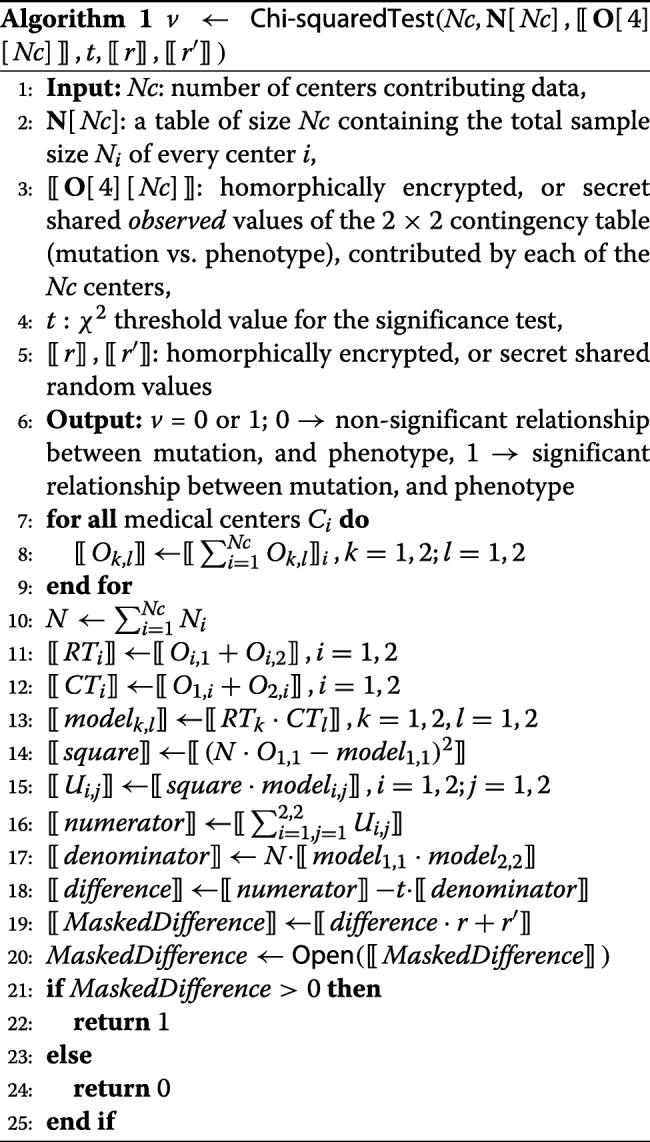


### Setup and security assumptions

#### Homomorphic encryption approach

**Fig. 1:**
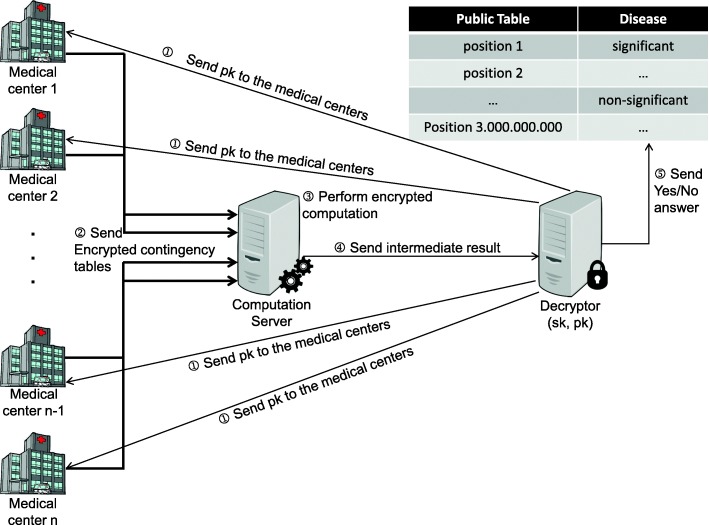
A schematic representation of the homomorphic scenario. Before the execution of the protocol, the decryptor generates a valid public and secret key pair for the homomorphic encryption scheme. Step ① of the protocol is to send the generated public key *pk* to all participating medical centers. Then, the medical centers compute their local contingency tables, encrypt them with the received public key, and send them to computation server in step ②. Step ③ is the actual secure computation of the encrypted, and masked *χ*^2^ value, which is then sent to the decryptor in step ④. By decrypting the masked *χ*^2^ value (using the secret key *sk*), the decryptor can only determine whether the result is significant or not, which is published in a public table in step ⑤

To solve the problem described in our application scenario with homomorphic encryption we need multiple parties, as indicated in Fig. 1. The steps of the process depicted in Fig. 1 are as follows. In the first step the decryptor will select the secret key, and associated public key for the homomorphic encryption, and make the public key available to all medical centers. Then all the medical centers will encrypt their contingency tables with the given public key, and send these encryptions to the computation server. Upon receiving all encrypted contingency table values, the computation server will first add them to construct the aggregated contingency table, and subsequently perform the operations of the Pearson *χ*^2^ test. Then, the computation server will send the result, which is masked with the technique we introduced in the “Efficient masking-based comparison” section, to the decryptor, who uses the secret key to decrypt the masked value, and performs the comparison.

It is important to note that in this model we trust the decryptor to decrypt the masked values, and post the corresponding correct yes/no value into the public table. Since the decryptor only decrypts masked values, the decryptor can only deduce the yes/no answer which will become public, anyway. No other information about the *χ*^2^ value is revealed to the decryptor. If the system would be adapted to reveal the actual *χ*^2^ value, the party receiving the encrypted result would first have to authenticate itself to make sure that it is a trusted entity (like a medical doctor, for example). If this authentication is considered insufficient by the medical centers contributing their data, they could still prevent the authenticated party from being a single point of trust by introducing a multiparty computation to perform the decryption based on a secret shared decryption key. The solution based on homomorphic encryption does rely on the following two security assumptions:

- The computation server is honest but curious: It will follow the stated protocol to provide the desired functionality, and will not deviate, nor fail to return the results. The computation server can however monitor the result of every operation it performs. This assumption is reasonable for an economically motivated cloud service provider. The cloud is motivated to provide excellent service, yet it would take advantage of extra available information.
- We want the decryptor to only decrypt the result of the masked comparison. He should not be allowed to see the input values, since he has the key to decrypt them. Therefore, we presume that the communication between the centers, and the computation server is hidden from the decryptor. This can be achieved by performing the communication over authenticated, secure channels. An alternative way to solve this is by introducing the multiparty computation for the decryptor. Each party only has a part of the decryption key, and hence will never be able to decrypt the values of the encrypted contingency table.

For the homomorphic evaluation of the *χ*^2^ statistic we use the FV scheme, introduced by Fan and Vercauteren [20]. Moreover, we base our implementation on the FV-NFLlib software library [21] in which the FV homomorphic encryption scheme is implemented using the NFLlib software library developed for performing polynomial arithmetic computations (as described by Melchor et al. [22], and released in [23].

#### Secure multiparty computation approach

To address the challenge of disease gene identification using secure multiparty computation techniques, in the setting described in our application scenario, we deploy MASCOT [24]. We selected MASCOT [24] as the most suitable multiparty computation solution, because it is currently the most efficient proposal, offering malicious static security with a dishonest majority. This means that any number of the computing parties may deviate from the protocol execution, and this will be detected without leaking information, other than what the correct protocol execution would reveal. Corruption may only occur prior to the beginning of the protocol execution, affecting up to *n*−1 (out of the *n*) computing parties.

For our multiparty computation approach, we first need to determine the number of computation servers *n* (*n*≥2) that we have at our disposal. Given that the underlying protocol offers security against any coalition of *n*−1 computation servers, we consider the security of the whole system to increase as the number of computation servers increases. However, the number of computation servers is inversely proportional to the efficiency of the solution. Therefore, we consider that three computation servers is an adequate number of servers, both from an efficiency/plausibility perspective, and from a security perspective. If any two of the three computation servers that we assume get compromised, or otherwise behave dishonestly, or even collude, the solution still guarantees input privacy, and does not accept incorrect results.

We assume a preprocessing phase that can take place offline, at any moment prior to the actual protocol execution. This is to create the necessary randomness for the medical centers to contribute their inputs in a secret shared manner to the computation servers. In addition, the preprocessing phase creates authenticated randomness to be used in the online phase, so as to boost the efficiency of computing multiplications on the shares, which requires interaction amongst the servers.

**Fig. 2:**
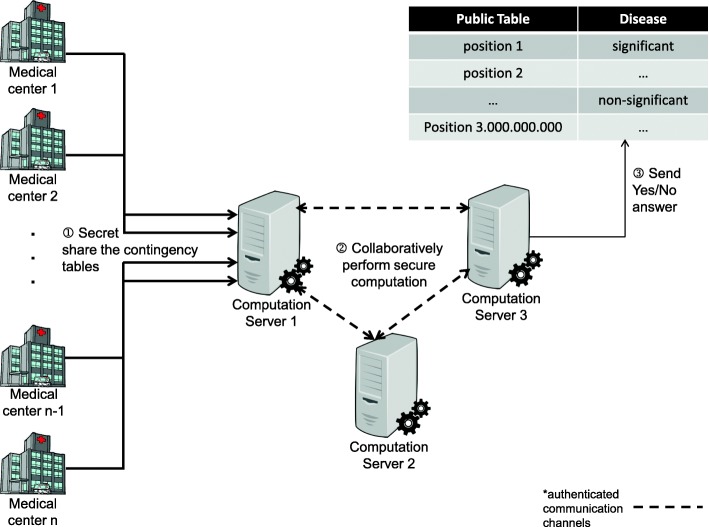
A schematic representation of the multiparty computation scenario. Any time prior to the protocol execution each of the medical centers computes their local contingency tables, and secret shares them to the three computation servers, as indicated in step ① of the protocol. In step ②, the computation servers securely compute the *χ*^2^ value, and perform a secure comparison to determine whether the value is significant or not. This reveals no information about the inputs, or the actual *χ*^2^ value to the individual computation servers. In step ③ the computation servers reconstruct the final result, which indicates significance or non-significance, by combining their individual secret shares, and they publish this result in the public table

The medical centers that wish to contribute their private inputs, first need to agree on a common format for this data (e.g., what is the order of sending the contingency tables). Then, they need to secret share their contingency tables to the three computation servers, which can also be pushed to an offline, preprocessing phase. Given that all contributing medical centers have shared their private contingency tables to the computation servers, the online phase starts. During the online phase the servers perform both local, and interactive secure computations, and they finally reveal per contingency table whether the relationship between a mutation at a certain DNA position, and a phenotype is significant or not, without disclosing further information on the underlying data. A schematic representation of this approach is presented in Fig. 2. Our protocol calculates the units of inequality (4), as well as the inequality check itself, using MASCOT [24], and its implementation of standard (bit-decomposition based) comparison.

In our setting, we consider the computing parties that actually execute our protocol (i.e., the computation servers), different from the parties contributing their inputs (i.e., the medical centers), as shown in Fig. 2. For the offline phase, together with the preparation of the randomness necessary for the execution of the online phase of the MPC protocol, we wish to perform the required preprocessing that will allow the medical centers to correctly contribute their inputs, without compromising privacy. To allow the medical centers to correctly and securely contribute their inputs, we use the *Output Delivery*, and *Input Supply* protocols proposed by Damgård et al. [25].

## Results

### Implementation and performance analysis

For both approaches, namely the homomorphic encryption approach, and the multiparty computation approach, we executed the program 10 times per case, and calculated the average execution time for our timing results. To evaluate the scalability of our protocols, we have considered the cases where our system receives data from 20, 40, 60, 80, and 100 medical centers, respectively. We assume each medical center to contribute data of 10000 subjects (i.e., the total number of subjects per case is 200000, 400000, 600000, 800000, and 1000000, respectively). Our timing results represent one SNP-phenotype combination (i.e., the computations needed for a single contingency table), and the proposed solutions scale linearly in the number of SNP-phenotype combinations.

#### Homomorphic encryption approach

In order to assess the practical performance, and verify the correctness of the selected parameters of the homomorphic scenario, we implemented the privacy-preserving *χ*^2^ computation. Our presented timings are obtained by running the implementation on a computer equipped with an Intel Core i5-4590 CPU, running at 3.30 GHz.

The encryption time does not depend on the number of centers, since the centers can perform the encryption in parallel. The measured encryption time for one contingency table is 17.1 ms. The time to decrypt the result will also not depend on the number of centers participating in the computation. The measured decryption time is 21.1 ms. The timings for the computation server are listed in Table 2, since these timings are dependent on the number of medical centers participating. These timings do not increase significantly for an increasing number of centers. Hence, we can conclude that considering CPU time, our solution scales really well for increasing number of medical centers participating in the computation.

**Table 2:** CPU time of the computation server for analyzing one SNP with the homomorphic solution using 1 CPU core

Centers	Patients	CPU time computation server
20	200,000	1.48 s
40	400,000	1.52 s
60	600,000	1.53 s
80	800,000	1.56 s
100	1,000,000	1.57 s

For the homomorphic setup, there is no communication cost during the computations. The communication cost comes from sending values from each of the three parties to the next. We have three points of communication: the public key has to be sent from the decryptor to the medical centers; the encrypted values of the contingency tables have to be sent from the medical centers to the computation server; and the result has to be sent from the computation server to the decryptor. The size of the public key that needs to be sent to the different medical centers is 186 kB. The data needed to send one contingency table to the computation server is 2.1 MB. The communication cost between the medical centers, and the computation server is the number of centers participating times the amount of data needed to send one contingency table. So this communication cost increases linearly with the number of centers contributing to the computation. We only have to send the resulting value from the computation server to the decryptor, which gives a communication cost of 0.54 MB.

#### Secure multiparty computation approach

We have built a proof of concept implementation of our MPC approach using the platform provided by Keller et al. [24] in SPDZ-2 [19]. We ran our experiments for timing the execution of our protocol on a desktop computer equipped with an Intel(R) Core(TM) i5-3570K processor, at 3.40GHz, with 16.00 GB RAM, and the Ubuntu 17.04 operating system.

We have only considered the online phase of the protocol, as the preprocessing is protocol-independent, and can be executed at any moment, well before the execution of the online phase. We note, however, that the offline phase is also practically efficient, and we refer the reader to Keller et al. [24] for more details on the throughput of the offline phase. Every time we recorded timings, before the execution of the online phase, we ran the setup script provided with the SPDZ-2 software. This script simulates the offline phase, and creates all the necessary randomness for the execution of the online phase. The fact that the offline phase is simulated does not affect the performance, or efficiency of the online phase.

Our experiments were conducted on localhost with three computation servers. Hence, we do not take the network latency into account in the timing results we report. We do present the size of the data that each server has to send, as well as the communication rounds, and we consider this information to be sufficient for the reader to calculate the additional communication cost, based on the available network bandwidth.

For all our timing results we have executed our protocol 10 times per case, and calculated the average execution time. The communication cost of the protocol is constant. For our experiments we have established that all input data is shared by one of the computation servers (namely Server 1), instead of the medical centers that would contribute the data in a real setting.

In Table 3 we present the execution times of our approach, as well as the data sent by Server 1, including the sharing of the original inputs. Recall, however, that the sharing of the inputs can be performed in a preprocessing phase, prior to the actual protocol execution, allowing the online phase to be less communication intensive. The timings for Server 1 are presented separately, because it has to do some extra tasks, such as sharing the inputs, collect all the final results, and print them, which is reflected in its execution times. The other two servers are grouped together, as their execution times are similar. The communication cost for Server 1 is analyzed in Table 3, while for the other two servers it is constant, and equal to 4.2 kB. The protocol completes its execution in 10 communication rounds.

**Table 3:** Performance of the MPC approach for analyzing one SNP using 1 CPU core (in each computation server)

		Server 1		Server *i*, *i*≠1
Centers	Patients	CPU time	Data sent	CPU time
20	200,000	2.2 ms	12.7 kB	1.9 ms
40	400,000	2.3 ms	17.8 kB	2.0 ms
60	600,000	2.3 ms	23.0 kB	2.0 ms
80	800,000	2.5 ms	28.1 kB	2.2 ms
100	1,000,000	2.4 ms	33.2 kB	2.1 ms

## Discussion

From the setup description of both suggested techniques, one can determine the first significant difference between them: in the homomorphic setting, the medical centers only have to encrypt, and send their data to one party, namely the computation server; while for the multiparty computation they have to secret share their data with two or more computation servers. The execution times resulting from our experiments show that the MPC approach is significantly faster than the homomorphic approach. Even if we assume the encryption of the contingency tables by the medical centers to be part of a preprocessing phase, the homomorphic approach will take more than a second to complete its execution, while the computations in the MPC setup take only a few milliseconds. In terms of communication cost, the homomorphic setup has the advantage that it needs no communication during the computations. However, in terms of total amount of data that has to be transferred between the different parties, the MPC setup outperforms the homomorphic setup once more. We therefore recommend the MPC approach, as it is the most efficient out of the two approaches, and it does not rely on the strong assumption of semi-honest parties participating in the protocol.

Having compared the HE, and MPC approaches in a setting addressing the exact same problem, we have established that MPC can provide more efficient solutions with more relaxed security assumptions. Thus, we plan to proceed with future work on computing state-of-the-art statistics used in GWASs (instead of the more simple *χ*^2^ test) in a privacy-preserving way, using MPC. To this end, we consider an interesting first step to study how we can express, or approximate logistic regression with low degree polynomials. Then, we can deploy MPC for computing them securely, which will yield solutions efficient enough to be used in practice.

## Conclusions

Our work shows that, as long as we can express the statistics to be calculated with low-degree polynomials, privacy-preserving GWAS has become practical. We made the first step to efficient privacy-preserving GWAS with the secure calculation of the *χ*^2^ test. Our solutions provide provable security guarantees, while being efficient for realistic sample sizes, and number of medical centers contributing data to the studies. Interestingly, our solutions scale logarithmically in the number of subjects contributing data to the study, which means that as GWAS population sizes grow, our approach will remain suitable. We also propose a new masking-based comparison method, and show that in certain application scenarios, such as the GWAS scenario at hand, comparisons can be executed efficiently even in the HE setting, without leaking useful information about the underlying data.

## Abbreviations

BGV: Brakerski Gentry Vaikuntanathan
CPU: Central processing unit
DNA: Deoxyribonucleic acid
FV: Fan Vercauteren
GHz: Gigahertz
GWAS: Genome-wide association study
HE: Homomorphic encryption
kB: Kilobytes
MAF: Minor allele frequency
MASCOT: Malicious arithmetic secure computation with oblivious transfer
MB: Megabytes
MPC: Multiparty computation
ms: Milliseconds
NFLlib: NTT-based fast lattice library
NGS: Next generation sequencing
NTT: Number theoretic transform
RAM: Random access memory
s: Seconds
SGX: Software guard extensions
SNP: Single nucleotide polymorphism
SNV: Single nucleotide variation (with SNV we will refer to both SNP and SNV)
SPDZ: Smart Damgard Pastro Zakarias
YASHE: yet another somewhat homomorphic encryption scheme

## Footnotes

1 Our method can be also extended to contingency tables of larger size.
